# Nanotechnology Innovations Transforming Oral Health Care and Dentistry: A Review

**DOI:** 10.7759/cureus.46423

**Published:** 2023-10-03

**Authors:** Rishika Dakhale, Priyanka Paul, Aparna Achanta, Kajal P Ahuja, Mrunal Meshram

**Affiliations:** 1 Public Health Dentistry, Sharad Pawar Dental College and Hospital, Datta Meghe Institute of Higher Education and Research, Wardha, IND; 2 Orthodontics, Sharad Pawar Dental College and Hospital, Datta Meghe Institute of Higher Education and Research, Wardha, IND; 3 Oral Medicine and Radiology, Sharad Pawar Dental College and Hospital, Datta Meghe Institute of Higher Education and Research, Wardha, IND

**Keywords:** nanomaterials, dentistry, nanoparticles, nanotechnology, nanorobots, nanodentistry

## Abstract

Nanotechnology has revolutionized dentistry by transforming how oral health care is conceptualized, delivered, and maintained. Harnessing nanomaterials and advanced clinical instruments has opened new avenues for precision and innovation across various aspects of dental care. Nanotechnology offers the potential for precise pain management, tooth restoration, and alleviating dental hypersensitivity. Nanomaterials can occlude exposed dentinal tubules, enhancing patient comfort and overall oral well-being. Orthodontic therapy is also revolutionized by nanomaterials with shape memory properties, enabling rapid and more efficient tooth movement. The development of groundbreaking products and therapeutic alternatives is supported by ongoing research efforts, enabling the formation of dental implants, fillings, and prosthetic devices that closely mimic natural tooth characteristics. Nano-delivery systems are being devised for precise drug delivery within the oral cavity, ensuring optimal therapeutic outcomes with minimal side effects. The integration of nanotechnology in dentistry represents a groundbreaking evolution beyond the conventional boundaries of oral health care, enabling the development of innovative diagnostic techniques and improved oral well-being.

## Introduction and background

The art and science of material engineering at scales smaller than 100 nanometers is known as nanotechnology [1]. By improving the mechanical and physical qualities of materials and facilitating the development of novel diagnostic techniques and nano-delivery systems, it revolutionized the fields of medicine and dentistry [2]. Due to the special properties of nanoparticles, research on it is still ongoing. A distinctive physical property of nanoparticles is their high surface-to-core ratio, which denotes that the surface of the nanoparticle contains more atoms than the inside of the particle [3]. This is particularly helpful because, in contrast to core atoms, surface atoms contain unbound surfaces and can form new powerful interactions. Therefore, nanoparticles are more reactive [4]. This makes it possible for them to be easily used and modified for various purposes.

Quantum effects can begin to dominate the properties of matter; as size is reduced to the nanoscale it can affect the optical, electrical, and magnetic behavior of materials, particularly as the structure or particle size approaches the smaller end of the nanoscale [5]. The mechanical properties of these enhanced nanoparticles are improved, including toughness, stiffness, transparency, scratch, and abrasion resistance. Modern technology has transformed dentistry and offers the ability to deliver full oral health care with the use of nanotechnology and its applications in dentistry [6]. New treatments are also being proposed, along with a plethora of innovative nanotechnology products. Nanotechnology has raised hopes for improved oral healthcare delivery and improved maintenance via ongoing research in the diagnosis, treatment, and prevention of oral disorders [7].

## Review

Methodology

**Figure 1:**
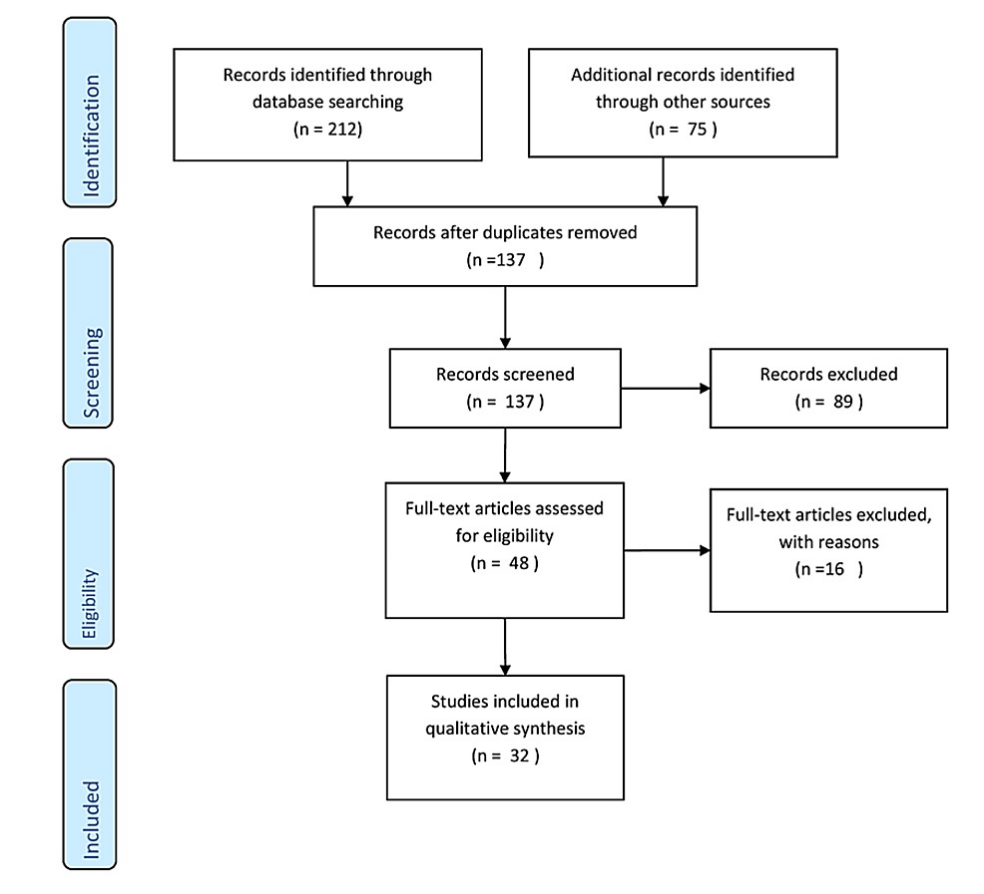
PRISMA flow diagram for search strategy. Adopted from PRISMA. PRISMA, Preferred Reporting Items for Systematic Reviews and Meta-Analyses.

This review carefully gathered literature on the application of nanotechnology in dentistry utilizing electronic databases such as PubMed, Scopus, and Web of Science. Keywords like "Nanomaterials," "Nanorobots," "Nanotechnology," "Nanoparticles," "Dentistry," and "Nanodentistry" were used to search the database. Articles on applications of nanotechnology in dentistry were included, but publications focusing on the application of nanotechnology in other disciplines were omitted. The inclusion criteria included relevant books, articles, studies, conference presentations, gray literature or unpublished literature, and reviews. The study selection procedure included screening titles and abstracts, followed by a full-text evaluation of relevant papers. The final group of included research offers a thorough analysis of the evidence that is currently available on the use of nanotechnology in dentistry. The results were combined and analyzed to draw meaningful conclusions. Figure 1 describes the selection process of articles used in our study.

Approaches to nanotechnology

There are five approaches to nanotechnology, which include top-down approach, bottom-up approach, functional approach, biomimetic approach, and speculative approach. In the top-down approach, particles are made smaller by grinding or milling and then the nanodimension is achieved by further miniaturization of the particles. In the bottom-up approach, smaller components are assembled to make complex structures that are synthesized at the atomic or molecular level. The functional approach emphasizes the production of nanoparticles for a specific use. The biomimetic approach uses biomolecules for applications in nanotechnology. The speculative approach emphasizes mainly on societal implications of nanotechnology [8].

Applications of nanotechnology in dentistry

The purpose of the dental specialty known as "nanodentistry" is to maintain optimal oral health using nanomaterials, biotechnology, and nanorobotics. Unique chemical, magnetic, and electro-optical characteristics of nanoparticles influence their solubility, bioactivity, and antibacterial activities. New treatments available in this sector include dentition renaturalization, long-term hypersensitivity treatments, artificial bones and teeth, local anesthesia, orthodontic realignments, oral health maintenance, tissue engineering, and medication delivery techniques. The use of nanotechnology in dentistry has a substantial impact on the field of clinical dentistry. Application of nanodentistry can be divided into two types, top-down approach and bottom-up approach. 

Top-down approach 

Application of Nanotechnology in Restorative Dentistry

To treat carious, missing, or broken teeth, a variety of materials can be used, including nanocomposites, nano-glass ionomer cement (GIC), nanoceramics, bonding agents, coating agents, and nanosolutions [9]. Nanocomposites are universal filling materials with outstanding mechanical qualities for restorations under occlusal stress. Nanocomposite contains nanofiller particles of two different types: nanometrics and nanoclusters. The advantages of nanocomposites are that they possess exceptional mechanical qualities including hardness and strength as well as outstanding esthetic qualities with good polish retention and minimal polymerization shrinkage [10]. Nano-GICs are created to fulfill a variety of dental needs by utilizing nanotechnology to deliver value-added qualities. Fluoride-releasing capacity is provided by this novel type of GIC, which was created utilizing bonded nanofillers and nanocluster fillers. It is suitable for many clinical indications and is great for general dentistry. Nano-GIC has good polish, esthetics, and high wear resistance. Nanoceramics have organically modified ceramic nanoparticles and have a polysiloxane backbone. These nano-ceramic particles are best defined as inorganic-organic hybrid particles, with the inorganic component siloxane and the organic component methacrylic blending all of the particles with a resin matrix [11]. The nanoceramic particle strengthening effect may be responsible for the good resistance to microcrack propagation. The nanoceramic particles either reflect or absorb the propagating fractures. Bonding agents (nanobonds) with a focus on nanotechnology include silica nanofillers in their nanosolutions to strengthen the bonds. In the oral environment, the nano-contact zone produces an enduring calcium molecule that improves binding and resists enzymatic destruction. By carefully dispersing nanoparticles and utilizing strengthened bonding processes, this development marks a paradigm shift in dental care and ensures long-lasting restorations. Coating agents contain light-activated nanosized fillers that can be employed as a composite coating, GIC, jacket crowns, and veneers [12]. The incorporation of nanofillers provides an excellent polish on the restorations, preventing discoloration and increasing abrasion and wear resistance. Nanosolutions feature nanoparticles that are uniformly scattered, extending their function as bonding agents. One-step applications characterize the new generation of bonding agents. The greatest binding strength and prevention of particle settling are provided by the uniformly distributed nanofillers [13].

Applications in Preventive Dentistry

Nanotechnology is important in dental biofilms because it provides insights into microbial interactions, demineralization, and remineralization equilibrium. With its great affinity for negatively charged side groups, new silver nanotechnology is effective against biofilms [14]. It attacks several places within the cell and disrupts cellular pathways, the cell wall, and protein synthesis. Traditional teeth polishing might result in surface roughness, which promotes biofilm growth. Ultra-fine tooth polishing, which causes nanoscale roughness measured in nanometers, provides a way out that reduces discoloration and dental restorations appear better. Ultra-fine polishing not only maintains oral hygiene by preventing bacterial attachment, but it also adds to the overall appeal of restorations, eventually improving the quality and visual allure of dental care outcomes [15]. Nanotechnology microscope with emerging deep probe detectors that use the electromagnetic spectrum can scan the human body and reveal hidden things such as deep-seated cancers and hidden dental cavities. This technology is powered by terahertz radiation, which is located on the spectrum between light and radio waves [16]. These detectors enable the non-invasive examination of tissues and substances by utilizing terahertz radiation. This breakthrough opens the door to improved medical diagnostics, enabling the detection of hidden health concerns such as tumors and dental cavities that would otherwise go undetected [17].

Applications in Prosthetic Dentistry

Polymethyl methacrylate is utilized to produce removable prostheses but has significant downsides, including poor strength and fracture resistance. Titanium oxide and iron oxide are two examples of nanostructuring materials that are added to polymethyl methacrylate to increase strength, esthetics, and antimicrobial qualities. Dentures and dental crowns made of nano zirconia ceramic have improved hardness and fracture toughness. Great corrosion resistance and translucency with increased fracture toughness are all presented by nanoceramic grains [18]. Impression material that has nanofillers and vinyl polysiloxanes creates a unique siloxane impression material that has better qualities than the traditional impression material used. This formulation has a lower viscosity, a smoother application, fewer voids, better handling qualities, and better detail capture. It also has increased tearing resistance, distortion prevention, and heat resistance, transforming impression materials and improving the practicality and quality of dental treatments. Nanocomposite denture teeth are an innovative advancement in dental prostheses. These teeth are made of nanocomposite resin with nano-sized filler particles that are uniformly scattered [19]. This new material has numerous advantages, including excellent polishability, stain and impact resistance, and a vibrant surface structure. It is available in a variety of colors to completely mimic natural tooth shapes and to accommodate varied setup processes. These denture teeth provide not only excellent esthetics but also multifunctional adaptability. In essence, they are the denture technology of the future, combining sophisticated materials and painstaking design for increased esthetics and durability in dental prostheses [20]. The application of nanotechnology to implants represents a revolutionary opportunity for increasing osseointegration and overall implant success. The inclusion of nanoscale hydroxyapatite and calcium phosphate deposits on implant surfaces is accomplished by nanotechnology. This distinctive surface alteration promotes osseointegration by boosting osteoblast activity, resulting in increased bone formation. Surprisingly, these nanotechnology-enhanced implants have demonstrated a significant 150% improvement in osseointegration efficacy, resulting in a decreased number of appointments and a faster treatment duration. Implant technology is advancing by utilizing nanoscale features, resulting in greater prognosis, faster healing, and more efficient dental implant treatments [21].

Applications in Periodontology

Nanobone graft materials can meet some requirements for treating bone defects, such as osteoconductivity, high porosity, and nanostructure, that can absorb natural proteins that osteoclasts destroy. For periodontal treatment, triclosan-loaded nanoparticles may function as nanodrugs. In the near future, it is believed that a variety of nanostructures, nanotubules, hollow spheres, as well as nanocomposites, are utilized as drug delivery systems. Laser plasma application for periodontia includes nanoscale titanium oxide particles with diameters ranging from 20 to 50 nanometers that have been integrated into gel-like emulsions for various dental treatments. Laser pulses generate optical breakdown, which results in shock waves, microabrasion, and collagen formation. This novel method, in conjunction with laser irradiation, represents a viable way to advance dental treatments, enhance healing, and reduce patient discomfort. Periodontal bone grafts and bone replacement materials offer a larger surface area than existing synthetic bone grafting materials, thanks to their micro- and nanoporosity, which allows for optimum bone regeneration [22]. The creation of "smart" periodontal materials using nanotechnology will help in bone repair and regeneration [23]. Biomaterials that are calcium phosphate filled offer improved handling characteristics and enhanced flow, and integrate well with the host bone. It aids in the development of cartilage as well as bone [24].

Applications in Oral Radiology

Nanophosphor scintillators are a game-changing technology for digital dentistry imaging, offering higher image quality while minimizing radiation exposure. These scintillators convert X-rays into visible light, producing high-quality images with lower radiation exposure. Nanoparticles also act as radio-opaque agents, ensuring radioopacity without impairing the basic features of materials. This novel application of nanotechnology improves dental imaging precision and safety by allowing for crisp, detailed images while lowering radiation exposure [25].

Applications in Orthodontics

Nanorobots have the potential to revolutionize dental procedures by allowing direct manipulation of periodontal tissues, enabling rapid and painless tooth adjustments. Stainless steel wires containing nanomaterials offer high corrosion resistance and deformability, making them a promising avenue for optimizing orthodontic treatments. This combination of nanorobotic potential and state-of-the-art materials could reshape dental care, enhancing efficiency and patient experiences [26].

Applications in Oral Surgery and Diagnosis of Oral Cancer

Cell surgery is becoming a reality because of advancements in surgical instruments for nano or cellular scales. Nanoneedles and nanotweezers are pioneering the field, allowing for accurate suturing and incisions at the cellular level. These cutting-edge instruments provide considerable advantages, particularly in tumor tissue procedures, and represent a paradigm shift in surgical capabilities, providing greater precision and effectiveness in medical treatments at the smallest sizes. Nanoneedles and nanotweezers are meant to open up the prospect of cell surgery in the near future. To execute incisions at the cellular level, suture needles are also being designed using nanosized stainless steel crystals. Particularly in tumor tissue surgery, selective cell manipulation, as well as surgery, carried out using instruments scaled at the molecular level will be advantageous. Nanorobots with active analgesic capabilities will be suspended in a colloidal solution in future nanodentistry. These moving nanorobots enter the gingiva of the patient and reach the pulp. Dentists can manage these to eliminate the tooth's sensations. After the treatments are finished, the dentist instructs to return all sensations and to leave the nerve pathways. Different types of nanoparticles can be used to deliver drugs and genes and probe DNA structures. Liposomes, inorganic nanoparticles (such as gold and magnetic nanoparticles), solid lipid particles, nanocrystals, polymer therapeutics like dendrimers, fullerenes, and nanospheres and nanocapsules are a few of them [27].

Salivary Diagnostics by Nanotechnology and Applications in Oral Pathology

**Figure 2:**
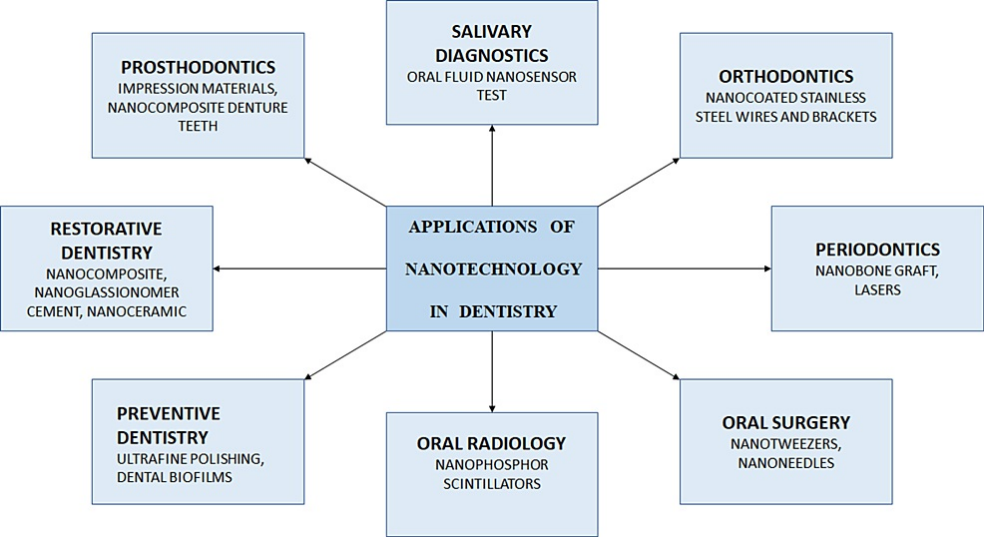
Applications of nanotechnology in dentistry as a top-down approach.

For the non-invasive and cost-effective diagnosis of oral disorders, saliva is a useful diagnostic tool. The electromechanical biosensors with excellent sensitivity and specificity for the diagnosis of oral illnesses can examine this medium. A portable, automated, user-friendly, integrated device called the oral fluid nanosensor test (OFNASET) can identify salivary proteins and nucleic acid targets. Figure 2 describes the applications of nanotechnology in dentistry as a top-down approach. Some of the materials utilized for cancer detection include nanoshells, carbon nanotubes, quantum dots, supermagnetic nanoparticles, nanowires, nanodiamonds, dendrimers, and freshly created nanosponges. It has the ability to deliver very toxic chemicals straight to the malignant cells and can identify even a single cancerous cell in vivo [28]. 

Bottom-up approach

Nanotechnology as a bottom-up approach includes major tooth repair and tooth renaturalization, hypersensitivity cure, orthodontic nanorobots, dental durability and cosmetics, nanorobotic dentifrice (Dentifrobots), halitosis, nanotech floss, photosensitizers, and carriers. With the use of genetic and tissue engineering, full new teeth might be grown in vitro for extensive tooth repair before being implanted in a socket [29]. The practice of "whole tooth renaturalization" might gain popularity and displace more common operational dental treatments. With the regeneration of tissues, the patient may benefit from replacing the missing tooth. The dentinal tubules can be selectively occluded by biological materials created using nanotechnology, which can treat the patient quickly and effectively. Within minutes of administration, they can ease the pain and anxiety associated with it [30]. Nanorobots can directly alter the periodontium, including the alveolar bone and periodontal ligament, causing the teeth to move quickly and painlessly in a few hours. It is possible to extend the life of teeth by exchanging the enamel layers with pure sapphire and diamond, which have high hardness and strength. Nanorobotic toothpaste may be marketed as toothpaste or mouthwash. Once a day, they move across the supra as well as subgingival surfaces of the tooth while eliminating any debris and associated organic matter. These might monitor the oral cavity's regular environment while detecting cariogenic bacteria in the biofilm [31]. Dentifrobots reduce oral malodor by changing bacterial metabolism. The unique nanostructure of dental tape enables the inclusion of flavors as well as medicine administration. These are found on the target cell's surface and, when triggered by ultraviolet radiation, release free oxygen radicals that damage the target cell [32]. A summary of all the articles included in this review is listed in Table 1.

**Table 1:** Summary of the articles included in the review

Author	Year	Findings
Naaz and Asghar [1]	2022	Nanotechnology is a rapidly progressing field.
Kanaparthy and Kanaparthy [2]	2011	Nanodentistry will make possible the maintenance of comprehensive oral health by employing nanomaterials, including tissue engineering and dental nanorobots.
Kah et al. [3]	2007	Diagnosis of cancer with the help of nanoparticles.
AlKahtani [4]	2018	Nano-applications in dental diagnostics and dental materials are addressed.
Khurshid et al. [5]	2015	Developing new materials using nanomaterials and enhancing the properties of the existing materials.
Saravana and Vijayalakshmi [6]	2006	Nanotechnology promises advanced diagnostics, targeted drug delivery, and biosensors.
Panchbhai [7]	2019	Several theoretical applications were suggested or assumptions were made speculating the use of nanotechnology in dentistry.
Rybachuk et al. [8]	2009	Applications of nanotechnology in dentistry.
Bayne [9]	2005	Future developments for major areas of synthetic biomaterials are considered for bonding systems, composites, curing, ceramics, and cement.
Slavkin [10]	1999	Applications of nanotechnology in dentistry.
Khosla [11]	2009	Applications of nanotechnology in dentistry.
Fioretti et al. [12]	2010	Use of nanostructured and functionalized multilayered films containing melanocortin peptides as a new active biomaterial for endodontic regeneration.
Huang et al. [13]	2007	Nanodiamond materials can serve as highly versatile platforms for the controlled functionalization and delivery of a wide spectrum of therapeutic elements.
Patil et al. [14]	2008	Molecular technology is destined to become the core technology underlying all of 21st-century medicine and dentistry.
Ahuja and Panchbai [15]	2022	Nanorobots in dentistry are expected to enhance accuracy, reproducibility, and reliability.
Schleyer [16]	2000	Use of nanodentistry in the upcoming years.
Lippert et al. [17]	2004	Surface rehardening through investigation of the nanomechanical surface with nanoindentation in human enamel.
Sujatha et al. [18]	2010	Nanorobotics in dentistry.
Chandak et al. [19]	2021	Use of nanodentistry in endodontics.
Mitra et al. [20]	2003	Use of nanotechnology in advanced dental materials.
Bhardwaj et al. [21]	2009	Role of nanotechnology in dental biofilms.
Kong et al. [22]	2000	Role of nanotechnology in the management of periodontal disease.
Shrestha et al. [23]	2009	Use of nanoparticles in dentinal tubules to improve root canal disinfection.
Yuan et al. [24]	2010	Application of nanotechnology.
Danelon et al. [25]	2015	The addition of 3% trimetaphosphate nano to a conventional toothpaste promotes an additional remineralizing effect of artificial caries lesions.
Eronat et al. [26]	2014	Use of nanofilled glass ionomer cement.
Gad et al. [27]	2016	Nanotechnology in prosthodontics.
Gupta et al. [28]	2015	Risks and benefits of nanotechnology.
Shvero et al. [29]	2015	Application of nanocomposite.
Stander and Theodore [30]	2011	Implications of nanotechnology.
Suzuki [31]	2004	Nanotechnology in prosthodontics for denture teeth.
Tomisa et al. [32]	2011	Nanotechnology for dental implants.

## Conclusions

Nanodentistry is an expanding field that incorporates nanotechnology into dentistry with the goal of improving oral health and dental care. It makes use of nanomaterials and nanorobots in producing dental materials, diagnosis, treatment planning, and tissue regeneration. Nanomaterials can make dental restorations more durable and lower the risk of infection. However, it is still in its early phases, and thorough research and clinical studies are required to prove the safety and efficacy of nanotechnology.
